# Macrophages-derived NRG-1 promotes angiogenesis after ischemic stroke via the Akt-mTOR pathway

**DOI:** 10.4103/NRR.NRR-D-24-01323

**Published:** 2025-06-19

**Authors:** Jie Chen, Bo Wang, Danyang Fan, Xi Chen, Lenv Gao, Yun Luo, Zhenhua Zhou

**Affiliations:** 1 Department of Neurology, The First Affiliated Hospital of Army Medical University, Army Medical University, Chongqing, China; 2 Department of Neurosurgery, The First Affiliated Hospital of Army Medical University, Army Medical University, Chongqing, China; 3 Department of Rehabilitation Medicine, First Affiliated Hospital of Gannan Medical University, Ganzhou, Jiangxi Province, China

**Keywords:** acute ischemic stroke, angiogenesis, Clot, ErbB4, fluorescence-activated cell sorting, immune cell, inflammation, macrophages, Neuregulin-1, neuroprotection

## Abstract

Acute ischemic stroke remains a significant health concern owing to the limited efficacy of current therapeutic options. In recent years, Neuregulin-1 has exhibited promising neuroprotective effects in cerebral ischemia. However, the sources and functions of Neuregulin-1 have not yet been fully understood, which hinders its translation and broad application. Here, we collected paired clot and peripheral blood samples from patients with acute ischemic stroke to determine the sources of Neuregulin-1. In addition, we established an *in vivo* transient middle cerebral artery occlusion mouse model to investigate the therapeutic effects of Neuregulin-1 and its underlying molecular biological mechanisms. We observed a significant elevation in serum Neuregulin-1 levels among patients with acute ischemic stroke that correlated with severity of neurological impairment and clinical outcome. Using single-cell sequencing, we identified Neuregulin-1-positive macrophages among peripheral blood mononuclear cells that produced Neuregulin-1 post-ischemia. In addition, Neuregulin-1 promoted repair of the infarcted area, alleviating neuronal and myelin damage and improving overall behavioral recovery in mice. We found that Neuregulin-1 may exert these neuroprotective effects by promoting angiogenesis in the infarct area, and that this effect is mediated by Akt/mTOR/VEGF-dependent signaling. Our findings suggest that peripheral macrophages are a source of Neuregulin-1 post-stroke. Neuregulin-1 exerts its neuroprotective effects by promoting angiogenesis via Akt/mTOR/VEGF-dependent signaling, showing promising clinical translation potential.

## Graphical abstract

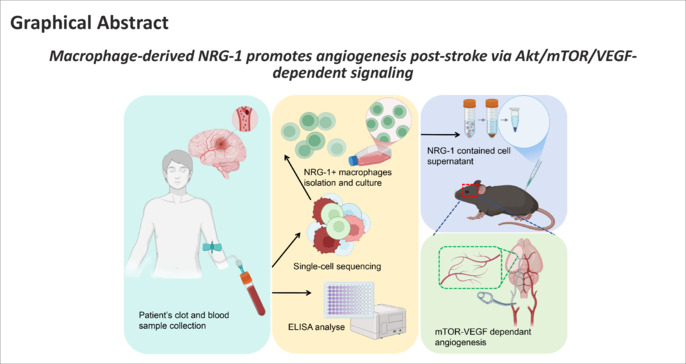

## Introduction

Stroke is a main reason for disability and mortality worldwide. It is estimated that approximately 80% of all newly diagnosed stroke cases are ischemic (Wang et al., 2017). The mechanisms of injury after acute ischemic stroke (AIS) are highly complex, but current international research generally focuses on the reperfusion of blood vessels and the reconstruction of blood flow after infarction. These injury repair mechanisms are crucial for the timely supply of oxygen and the timely removal of harmful substances and inflammatory factors. Currently, two primary reperfusion therapies are used for treating hyperacute ischemic stroke: interventional endovascular treatment and intravenous thrombolysis (Mead et al., 2023). Although stroke mortality has significantly declined, postoperative neuroprotection remains essential for improving patient outcomes. Consequently, neuroprotective therapies are gaining increased attention to address the unmet needs of patients after reperfusion (Liu et al., 2009; Wang et al., 2022).

Despite extensive research, neuroprotective therapies remain limited in scope and clinical application, particularly in neurology. The complexity of ischemic injury mechanisms contrasted with the lack of effective neuroprotective agents highlights the pressing need for innovative strategies to enhance patient outcomes. Recent research has highlighted angiogenesis in post-stroke recovery, sparking significant interest in pro-angiogenic agents (Fang et al., 2023). Therefore, effective pro-angiogenic factors are potentially significant treatment options. Angiogenesis, which is an essential protective mechanism following ischemia, promotes neuronal repair and functional recovery (Manoonkitiwongsa et al., 2001; Chen et al., 2025). The search for effective pro-angiogenic agents has been a key focus among neuroscientists, as they have the potential to significantly enhance stroke rehabilitation and improve patient quality of life.

Neuregulin-1 (NRG-1), a neuroregulatory protein primarily expressed in the central nervous and cardiovascular systems, plays a pivotal role in pathologies of the central nervous system, including neuropsychiatric disorders, neurodegenerative diseases, and cerebrovascular pathologies (Falls, 2003). NRG-1, a member of the epidermal growth factor family, regulates cell growth, differentiation, and survival by interacting with ErbB family receptors. Its epidermal growth factor-like domain facilitates receptor binding, while alternative splicing generates isoforms with distinct functions and tissue distributions (Berrocal-Rubio et al., 2024). Notably, it exhibits significant potential in mitigating neuronal damage and facilitating repair processes following injury. A previous study established the involvement of NRG-1 in neuropathological processes, particularly in neuropsychiatric disorders (Mei and Xiong, 2008). In addition, NRG-1 has demonstrated therapeutic potential in Alzheimer’s disease by mitigating cognitive impairment (Ou et al., 2021). Emerging evidence indicates that NRG-1 also provides neuroprotection following cerebral ischemia by lowering inflammation, alleviating neuronal death, and promoting oligodendrocyte proliferation and remyelination via activation of the ErbB-AKT signaling pathway (Xu et al., 2005; Noll et al., 2019; Cui et al., 2023). Additionally, transcriptomic analyses of cerebral endothelial cells under oxygen–glucose deprivation conditions revealed significant alterations in this pathway, alongside notable involvement of the AKT-mTOR signaling pathway (Luo et al., 2024). Our earlier study of patients with Moyamoya disease indicated an association between elevated serum NRG-1 levels and abnormal vasculature development in the skull and dura mater, suggesting its potential role in intracranial angiogenesis (Chen et al., 2024). Nevertheless, the specific role of NRG-1 in angiogenesis following cerebral ischemia has been largely unexplored.

The aim of this study was to elucidate the neuroprotective effects of NRG-1, particularly focusing on its role in promoting angiogenesis post AIS. Using single-cell sequencing, we investigated the association between NRG-1 and peripheral immune cells following cerebral ischemic stroke. Additionally, we explored the function of NRG-1 in facilitating angiogenesis in the post-ischemic brain to advance understanding and clinical translation of this promising neuroprotective agent.

## Methods

### Human sample collection and processing

For our study, the sample size was determined using G*Power software (version 3.19, Düsseldorf, DE), with an effect size of 0.6, an error probability (α) of 0.05, and a desired power (1 – β) of 0.8. Based on these parameters, the calculated minimum sample size required was 20 participants per group, with a noncentrality parameter (δ) of 2.62, a critical *t*-value of 1.73, degrees of freedom (*df*) of 18.10, and an actual power of 0.8 (**Additional Figure 1**). The study enrolled 20 adult patients with AIS and 23 healthy controls (recruited by advertisement) from Southwest Hospital, Third Military Medical University (Army Medical University) between January and May 2021. Diagnosis of AIS was established on the basis of following criteria (Yew and Cheng, 2015): 1) clinical manifestations (including focal neurological deficits including weakness, numbness, difficulty speaking, or other symptoms); 2) radiological findings including computed tomography (CT) scan and MRI; and 3) laboratory tests, including blood tests and cardiac evaluation. The inclusion criteria were as follows: 1) diagnosis of AIS, excluding those with stroke mimics; 2), complete in-hospital laboratory and follow-up data; and 3) the absence of autoimmune diseases or hematological disorders at discharge. The exclusion criteria were as follows: 1) patients diagnosed with stroke mimics; 2) incomplete in-hospital laboratory and follow-up data; and 3) diagnosis with autoimmune diseases or hematological disorders at discharge. We fully reviewed the demographic characteristics and medical histories of patients with AIS and healthy controls (**Table 1**). In addition, neurologic deficit was evaluated using the National Institutes of Health Stroke Scale (NIHSS) score (Adams et al., 1999), and stroke classification was confirmed by Trial of Org 10172 in Acute Stroke Treatment (TOAST) criteria (Adams et al., 1993; Lyden, 2017). Additionally, long-term neurological assessment was graded by the modified Rankin Scale (mRS) (van Swieten et al., 1988), with a favorable outcome defined as mRS ≤ 2, and mRS ≥ 3 defined as a poor outcome. Prior to treatment, 3–5 mL of peripheral whole blood sample was collected from both healthy controls and patients AIS, with written informed consent provided. This study was approved by the Ethics Committees of the First Affiliated Hospital of Army Medical University ((B)KY2021163), 14/11/2022) and conducted in accordance with the *Declaration of Helsinki*.

**Figure 1:**
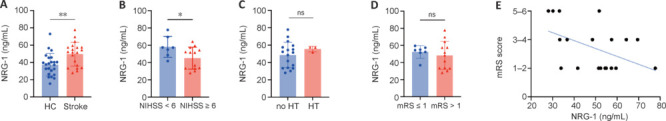
Serum NRG-1 levels are increased in patients with acute ischemic stroke and are related to neurological dysfunction severity. (A) Serum NRG-1 levels in patients with AIS and healthy controls. (B) Serum NRG-1 levels in patients with AIS with an NIHSS score ≥ 6 or an NIHSS score < 6 at admission. (C) Serum NRG-1 levels in patients with and without HT. (D) Long-term post-ischemia outcomes in patients with AIS with mRS ≤ 1 and those with mRS > 1. (E) Serum NRG-1 level is negatively correlated with 3-month mRS score (*r* = −0.449, *P* = 0.047). Data are indicated as mean ± SEM. Statistical significance was determined using Student’s *t*-test and Spearman correlation analysis; *P < 0.05; **P < 0.01; ***P < 0.001. HC: Healthy control; HT: hemorrhagic transformation; mRS: modified Rankin Scale; NIHSS: National Institutes of Health Stroke Scale; NRG-1: Neuregulin-1; ns: not significant.

**Table 1:** The clinical characteristics of AIS patients and healthy controls

	AIS (*n* = 20)	Healthy controls (*n* = 23)	*P*-value
Age, yr	62.5±9.1	60.1±7.3	0.347
Male, *n* (%)	14 (70)	14 (61)	0.519
Risk factors, *n* (%)			
Hypertension	13 (65.0)	9 (39.1)	0.165
Diabetes mellitus	6 (30.0)	3 (13.0)	0.323
Hyperlipemia	8 (40.0)	6 (26.1)	0.515
Smoking	11 (55.0)	8 (34.5)	0.306
Drinking	11 (55.0)	7 (30.4)	0.187
History of ischemic stroke	4 (20)	1 (4.3)	0.263
TOAST classification, *n* (%)		NA	NA
Large artery	9 (45.0)		
Cardioembolism	4 (20.0)		
Others	7 (35.0)		
Hemorrhage transformation, *n* (%)	3 (15.0)	NA	NA
NIHSS, *n* (%)		NA	NA
< 6	5 (25.0)		
≥ 6	15 (75.0)		
NRG1 (OD value)	1.14±0.29	0.89±0.27	0.005

AIS: acute ischemic stroke; NA: not applicable; NIHSS: National Institutes of Health Stroke Scale; NRG-1: Neuregulin-1; TOAST: Trial of Org 10172 in Acute Stroke Treatment.

### Enzyme-linked immunosorbent assay for Neuregulin-1

Serum NRG-1 was detected using a human NRG-1 enzyme-linked immunosorbent assay kit (KE00108, Proteintech, Wuhan, China) in strict accordance with manufacturer’s instructions.

### Establishment of the transient middle cerebral artery occlusion model

C57BL/6J male mice (6–8 weeks old, 22–25 g) were obtained from Beijing Vital River Laboratory Animal Technology Co., Ltd., China (license No. SCXK (Jing) 2021-0011)). Transient middle cerebral artery occlusion (tMCAO) was performed as described in previous studies (Wang et al., 2021; Huang et al., 2024). Mice were initially anesthetized in a chamber containing 3% isoflurane, followed by maintenance anesthesia with 1.5%–2.5% isoflurane (Cat# R510-22-10, RWD, Shenzhen, China). The entire procedure was performed on a temperature-controlled pad that was maintained at 37 ± 5°C. The neck muscles were exposed by blunt dissection, and the common carotid artery, internal carotid artery, and external carotid artery (ECA) were isolated. Next, the proximal part of common carotid artery and the distal end of ECA were ligated. Then, an incision was made in the ECA. Subsequently, a filament (1800A, Jialing Biotechnology, China) was inserted into the incision in the ECA and advanced to the MCA opening in the anterior cerebral artery through the common carotid artery to block left MCA blood flow. The filament was left in place for 1.5 hour to maintain cerebral ischemia and then removed. Successful establishment of the tMCAO model was defined as a 70% reduction in blood flow, as determined by Laser Speckle Imaging analysis (RWD). The mice in the sham group were subjected to the same tMCAO surgery without artery occlusion. Thirty mice (6–8 weeks old) were randomly divided into three groups: sham, tMCAO + vehicle, and tMCAO + NRG-1. Mice in the tMCAO + NRG-1 group were injected with supernatant containing NRG-1, from cultured NRG-1+ macrophages via the tail vein 1 hour after reperfusion. Mice in the tMCAO + vehicle group were injected with supernatant from NRG-1^+^ macrophages in the same manner. The mice were treated once daily for 7 consecutive days and sacrificed 14 days after injury to evaluate the effects of NRG-1 on long-term neurological deficits after tMCAO. All animal experiments were conducted with the approval of the Animal Ethics Committee of the Army Military Medical University (Approval No. AMUWEC20224297, February 28, 2022) and in strict accordance with the National Institutes of Health Guide for the Care and Use of Laboratory Animals (8^th^ ed., National Research Council, 2011).

### Fluorescence-activated cell sorting of Neuregulin-1-expressing macrophages

Peripheral whole blood from patients with stroke was collected in EDTA anticoagulation tubes at room temperature. First, 1.5 mL of peripheral blood was mixed with 1.5 mL of 1X PBS. Then, 3 mL Ficoll solution (Cat# 17-1440-03, GE Healthcare, Chicago, IL, USA) was carefully layered at the bottom of a 15-mL centrifuge tube, and the PBS–peripheral blood mixture was gently layered on top of the Ficoll. Next, the sample was centrifuged at room temperature at 2000 × *g* for 30 minutes with acceleration set to 1 and deceleration set to 0. Subsequently, the intermediate buffy coat layer was carefully transferred to a 15-mL centrifuge tube and then washed with 5 mL of fluorescence-activated cell sorting (FACS) buffer. The cells were stained with Zombie dye (Cat# 423113, BioLegend, San Diego, CA, USA) at a 1:300 dilution for 10 minutes at 4°C. Subsequently, to prevent nonspecific binding, Fc receptors were blocked using Fc blocker (Cat# 422302, BioLegend) at a 1:300 dilution for 10 minutes at 4°C. The cells were then incubated with PE/Cyanine7 anti-CD45 (Cat#368532, BioLegend) at a 1:300 dilution, APC anti-CD14 (Cat# 982506, BioLegend) at a 1:300 dilution, and FITC anti-NRG-1 (Cat# CL48866429, Proteintech) at a 1:50 dilution for 30 minutes at 4°C. After washing and resuspending in FACS buffer, Zombie–CD45^+^CD14^+^NRG-1^+^ cells were sorted using a FACSAriaII flow cytometer (BD, Franklin Lakes, NJ, USA).

### Preparation of cell suspensions from human clots and peripheral blood monocyte cells

Clots: Fresh clot samples collected from patients with AIS receiving endovascular treatment were placed on ice and processed in the laboratory within 2 hours. First, the clots were washed with precooled PBS to thoroughly remove visible blood. Then, each the sample was mechanically dissected into small pieces with a sterile scalpel. Next, the pieces were placed in a 5-mL tube (Cat# 0030119401, Eppendorf, Hamburg, Germany) with digestion cocktail containing 0.3 mg/mL DNase I (Cat# D7291-2MG, Sigma-Aldrich, St. Louis, MO, USA), 1 mg/mL collagenase P (Cat#11088882001, Roche, Basel, Switzerland), and 0.3 mg/mL DNase XII (Cat# D7291-2MG, Sigma-Aldrich) in DMEM medium and incubated at 37°C for 20 minutes. Afterwards, the homogenate was filtered through a 70-mm strainer and centrifuged at 500 × *g* for 10 minutes at 4°C. Subsequently, the red blood cells were lysed with 2 mL ACK Lysis Buffer (Cat# RT122-02, Tiangen, Beijing, China) at room temperature (RT) for 5 minutes. Then, 6 mL precooled PBS was added to neutralize the lysis buffer, and the mixture was centrifuged at 500 × *g* for 10 minutes at 4°C. After washing with precooled PBS containing 20 mM EDTA and 1.5% FBS, the cells were ready for staining and flow cytometry sorting.

Peripheral blood mononuclear cells (PBMCs): Initially, 1 mL of peripheral whole blood from patients with AIS was mixed with 1 mL PBS. Then, 3 mL Ficoll solution (Cat# 17-1440-03, GE Healthcare, Chicago, IL, USA) was carefully layered at the bottom of a 15-mL centrifuge tube, and the PBS–peripheral blood mixture was gently layered on the top. Next, the sample was centrifuged at room temperature at 2000 × *g* for 30 minutes with acceleration set to 1 and deceleration set to 0. Ficoll-enriched cells were collected for staining and sorting.

Single-cell RNA sequencing (scRNA-seq): PBMCs and digested clots from patients with AIS were first stained with Zombie dye (Cat#423113, Biolegend) for 10 minutes at 4°C. After treatment with a human Fc receptor blocker (Cat#422302, Biolegend) at 4°C for 15 minutes, the cells were then stained with anti-CD45 for 30 minutes at 4°C. Live human clot immune cells and PBMCs were sorted separately by gating on Zombie-CD45^+^ single cells. A Chromium Single Cell 30 Library and Bead Kit v.2 (10x Genomics) was used to generate scRNA-seq libraries, as previously described (Wu et al., 2020). In brief, single-cell gel beads were generated using 10,000 to 20,000 live cells in an emulsion. After reverse transcription, the gel beads in the emulsion were disrupted. The barcoded complementary DNA was then isolated and amplified via polymerase chain reaction (PCR). Subsequently, fragmentation, end repair, and A-tailing were performed, and sample indices were added during index PCR. The purified libraries were sequenced using a NovaSeq 6000 System (Illumina, San Diego, CA, USA; Liu et al., 2023).

### Quantitative reverse transcription–polymerase chain reaction

Total RNA from the brains of affected mice was extracted using TRIzol (Vazyme Biotech Co., Ltd, Nanjing, China). Subsequently, mRNA reverse transcription was performed using a cDNA Synthesis Kit (Cat# DP451, Tiangen) according to the manufacturer’s instructions. The cDNA was used as the template for quantitative reverse transcription–polymerase chain reaction (qRT-PCR) with SYBR-green Premix Ex Taq^TM^ (Cat# RR820A, Takara Bio Inc., Shiga, Japan) on a Bio-Rad CFX manager PCR system (V.1.5, Hercules, CA, USA). Gene expression was normalized to the sham group. **Table 2** lists the primers used for qRT-PCR. The reaction conditions were as follows: an initial denaturation step at 95.0°C for 30 seconds; 40 cycles of denaturation at 95.0°C for 5 seconds, annealing at 60.0°C for 30 seconds, and then in melt curve stage; and an indefinite hold at 4.0°C.

**Table 2:** Primer used for quantitative reverse transcription–polymerase chain reaction

Target gene	Sequence (5'–3')
*Tnf-α*	F: CCC TCA CAC TCA GAT CAT CTT CT
	R: GCT ACG ACG TGG GCT ACA G
*Il-1β*	F: GCA ACT GTT CCT GAA CTC AAC T
	R: ATC TTT TGG GGT CCG TCA ACT
*Il-10*	F: GCT CTT ACT GAC TGG CAT GAG
	R: CGC AGC TCT AGG AGC ATG TG
*Il-4*	F: GGT CTC AAC CCC CAG CTA GT
	R: GCC GAT GAT CTC TCT CAA GTG AT
*P65*	F: CTT CTG GGC CTT ATG TGG AGA TC
	R: GGT CCT GTG TAG CCA TTG ATC TT
*Erbb4*	F: CCT TCC TGC GGT CTA TCC GA
	R: CCA AAG TTG CCA TCT TTC CTG TA
*Akt*	F: ATG AAC GAC GTA GCC ATT GTG
	R: TTG TAG CCA ATA AAG GTG CCA T
*Mtor*	F: CAG TTC GCC AGT GGA CTG AAG
	R: GCT GGT CAT AGA AGC GAG TAG AC
*Actb*	F: ACT GTC GAG TCG CGT CC
	R: CTG ACC CAT TCC CAC CAT CA

Actb: β-Actin; F: forward; IL: interleukin; R: reverse; TNF-α: tumor necrosis factor-α.

### Western blotting

Western blotting was performed as described previously (Wang et al., 2023). First, total protein from peri-ischemic mouse brain tissue was extracted with RIRA Lysis Buffer (Cat# P0013B, Beyotime, Shanghai, China). Next, the protein concentrations were detected using a Bicinchoninic Acid kit (Cat# P0009, Beyotime). After mixing with 5× loading buffer (Cat# P0015, Beyotime), the protein samples were separated electrophoretically on SDS-polyacrylamide gels (Cat# PG112, Epizyme, Shanghai, China) and subsequently wet-transferred onto polyvinylidene fluoride (PVDF) membranes (Cat# ISEQ07850, Roche). The protein-carrying PVDF membranes were blocked with 5% non-fat dry milk for 2 hours at RT and sequentially immersed in primary antibody overnight at 4°C. The antibodies used were as follows: mouse anti-GAPDH (1: 5000, Cat# 60004-1-Ig, Proteintech); mouse anti-p65 (1:1000, Cat# sc-8008, Santa Cruz Biotechnology, Santa Cruz, CA, USA); mouse anti-p-p65 (1:1000, Cat# sc-135769, Santa Cruz Biotechnology); mouse anti-tumor necrosis factor-α(1:1000, Cat# sc-52746, Santa Cruz Biotechnology); mouse anti- interleukin (IL)-1(1:1000, Cat# sc-32294, Santa Cruz Biotechnology); rabbit anti-IL-4 (1:1000, Cat# 66142-1-Ig, Proteintech); rabbit anti-IL-10 (1:1000, Cat# 60269-1-Ig, Proteintech); mouse anti-ErbB4 (1:1000, Cat# sc-71071, Santa Cruz Biotechnology); mouse anti-p-ErbB4 (1:1000, Cat# sc-81491, Santa Cruz Biotechnology); rabbit anti p-AKT (1:2000, Cat# 4060, CST, Danvers, MA, USA) rabbit anti-AKT (1:1000, Cat# 9272, CST); rabbit anti-p-mTOR (1:1000, Cat# 5536, CST); and rabbit anti-mTOR (1:1000, Cat# 2972, CST). Next, the PVDF membranes were incubated with secondary antibody for 2 hours at RT. Finally, the protein bands were detected using an enhanced chemiluminescence detection reagent (Cat# WP20005, Thermo Fisher Scientific, Waltham, MA, USA), and the band density was quantified using an imaging system (Evolution-Capt Edge, Vilber, France).

### Assessment of cerebral infarct volume and neurological function

2,3,5-Triphenyltetrazolium chloride (TTC) (Cat# T8170, Solarbio, Beijing, China) staining was used to determine cerebral infarct volume. Briefly, mice were sacrificed with deep isoflurane anesthesia, and the brain tissue was gently extracted. Then, brains were frozen at –20°C for 20 minutes before being cut into 1- to 2-mm-thick coronal slices. Next, the brain slices were fully immersed in 2% TTC staining solution at 37°C for 10 minutes. Finally, the coronal brain slices were fixed with 4% paraformaldehyde and photographed. ImageJ software (version 1.8, National Institutes of Health, Bethesda, MD, USA) was used to quantify the infarct volume, as follows: (area of the contralateral hemisphere minus the non-infarcted area of the ipsilateral hemisphere)/(area of the contralateral hemisphere × 2) × 100%.

Short-term neurological deficits in mice subjected to ischemic stroke were detected using the Longa and Garcia tests (Guan et al., 2024). In addition, long-term neurological performance was assessed using the open field test 1, 3, 7 days after tMCAO, as described previously (Wang et al., 2023; Kang et al., 2024). All behavioral tests were carried out in a double-blind manner.

### Immunofluorescence staining

Immunofluorescence (IF) staining was carried out as described previously (Wang et al., 2023). In brief, mouse brains were fixed with 4% paraformaldehyde overnight at 4°C. Subsequently, the fixed brains were dehydrated in a 15% sucrose solution at 4°C until they sunk then transferred to a 30% sucrose solutions for 2–3 days. Next, the dehydrated mouse brain tissue was embedded in Optimal Cutting Temperature (OCT) compound, frozen at –20°C, and sliced into 18-µm coronal sections using a Leica cryostat microtome (CAM1860, Leica, Wetzlar, Germany). To assess angiogenesis, we administered BrdU 50 mg/kg once daily for 7 days. For IF staining, the coronal brain slices were washed with phosphate-buffered saline (PBS) solution three times for 5 minutes each time. Next, to avoid non-specific staining, the slices were incubated with blocking buffer (Cat# P0102, Beyotime) for 1.5 hours at room temperature. Then, the slices were immersed in rabbit anti-CD31 (1:100 dilution, Cat# 557355, BD, Franklin Lakes, NJ, USA) and Alexa Fluor 488-labeled mouse anti-BrdU (1:200 dilution, Cat# HA720186F, HuaAn, Hangzhou, China) for 16–24 hours at 4°C, washed with PBS three times, and incubated with goat anti-rabbit 594 (Cat# A-11035, Thermo Scientific, Waltham, MA, USA) for 2 hours at RT. In addition, the cell nuclei were stained with Hoechst 33342 (Cat# YA2929782, Thermo Scientific). Finally, the IF was observed using a LSM900 microscope (Zeiss, Jena, DE). ImageJ was used to perform quantitative analysis of CD31- and BrdU-positive spots.

### Immunohistochemical staining

Immunohistochemical (IHC) staining was also performed as described previously, with some modifications (Liu et al., 2023; Ye et al., 2023). Briefly, formalin-fixed and paraffin-embedded mouse brain tissue was cut into 4-µm-thick serial coronal slices. After deparaffinization and dehydration, the slices were blocked with streptavidin peroxidase at 37°C for 30 minutes. Next, they were immersed in antigen epitope retrieval citrate buffer and heated at high pressure for 150 seconds. Then, the slices were blocked with goat serum (Cat# 19G22C09, BOSTER, Wuhan, China) for 1 hour at 37°C and immersed in an anti-CD31 antibody (1:200 dilution, Cat# ab28364, Abcam, Cambridge, UK) for 16–24 hours at 4°C. Subsequently, the slices were washed three times with 1×PBS for 5 minutes each time and immersed in a goat anti-rabbit antibody (Cat# K5007, Dako, Copenhagen, DK) for 1 hour at 37°C. A 3,3′-diaminobenzidine (DAB) solution (1:100 dilution, Cat# K5007, Dako) was applied, and the target protein was visualized under a microscope (KF-PRO-005-EX, KFBIO, Ningbo, China) within 1–2 minutes. The cells were counterstained with hematoxylin, and a digital pathology slide scanner was used to obtain the images. ImageJ was used to quantify the CD31-positive regions.

### Transmission electron microscopy

Transmission electron microscopy (TEM) was used to measure the integrity of the white matter bundle, as we previously described. Within 2 minutes of brain tissue harvesting, samples (1 mm^3^) were immersed in 1.25% glutaraldehyde for 3 days at 4°C. Next, the samples were rinsed three times with PBS and further fixed with 1% osmium tetroxide (OsO4) for 2 hours. Then, the samples were embedded in epoxy resin and cut into 50-nm-thick serial slices using a Leica ultra-microtome (EM UC7, Leica, Wetzlar, Germany). Images were obtained by TEM (HT7700, Hitachi, Tokyo, Japan). The G-ratio was measured by ImageJ to evaluate demyelination in each field. Fields were randomly chosen from each white matter bundle, and over 25 white matter bundles from each sample were assessed and included in the calculations.

### Nissl staining

Nissl bodies are vital indicators used to measure the neural structure of the brain. The number of Nissl bodies decreases significantly when neurons are stimulated. Changes in the number of Nissl bodies closely reflect changes in cerebral neuron morphology. Nissl staining was performed as described previously (Cat# C0117, Beyotime) (Rui et al., 2021). Paraffin-embedded coronal brain sections were deparaffinized and dehydrated, then incubated in Nissl staining solution (Cat# C0117, Beyotime) for 10 minutes at RT. Next, these sections were dehydrated by immersion in 75% alcohol for 2 minutes and mounted with Permount TM Mounting Medium (Kanto, Tokyo, Japan). Images were obtained using a microscope (KFBIO, KF-PRO-005-EX, Ningbo, CHN).

### Laser speckle contrast imaging

Laser speckle imaging was used to assess cerebral blood flow (CBF) in mice. Briefly, C57BL/6J mice were deprived of food and water for 6 hours and then placed in the laser speckle imaging device and allowed to acclimate for 30 minutes. After being anesthetized with isoflurane, the mice were placed on a heating pad to maintain body temperature, and their breathing was closely monitored. Using a dental drill and surgical instruments, a bone window is opened, and the mouse was secured on the imaging system (RWD) platform. The laser was turned on and directed at the bone window, and the speckle signal was captured and recorded through a camera (RWD). Subsequently, data analysis and processing were performed.

### Analysis of single-cell sequencing data from the GEO database

Single-cell sequencing data from the brain tissue of mice subjected to tMCAO were retrieved from the GEO database (GSE174574) and analyzed using R language (v.4.3.3, Auckland, New Zealand).

### scRNA-seq data analysis

Seurat (version 2.0, Robert Gentleman and Ross Ihaka, Boston, MA, USA) was used to analyze patient data that were acquired by barcode counting, filtering, and unique molecular identifier counting using Cell Ranger (v.2.1.0, MA, USA). Only cells with a percentage of mitochondrial genes below 0.05% were included. Cells were excluded from the downstream analysis if the number of detected genes was in the top 0.2% or the bottom 0.2%. Unique molecular identifier (UMI) counts were normalized to the UMI count per million total counts and subsequently log-transformed. Variable genes were selected based on average dispersion and expression, and t-SNE or U-MAP visualizations were constructed based on the selected principal component analysis dimensions. Marker genes were identified using the Seurat function FindAllMarkers. Heatmaps were generated based on scaled marker gene expression. Kyoto Encyclopedia of Genes and Genomes (KEGG) analysis was used to identify enriched biological pathways and molecular functions associated with the gene set.

### Statistical analysis

Data are shown as mean ± SEM. Graphs were generated using GraphPad Prism 7.0 (GraphPad Software, San Diego, CA, USA, www.graphpad.com). The counterpart size was calculated using G*Power software (version 3.19, USA). Spearman correlation analysis was used to analyze the association between serum NRG-1 and clinical features. Depending on the dataset, statistical analysis was performed by unpaired Student’s *t*-test or one-way analysis of variance with Tukey’s *post hoc* test. Statistical significance was set at *P* < 0.05.

## Results

### Serum Neuregulin-1 levels are elevated in patients with acute ischemic stroke and correlate with neurological dysfunction severity

To assess the predictive value of serum NRG-1 in AIS patients, we collected blood samples from 23 healthy subjects and 20 patients with AIS. **Table 1** shows the baseline characteristics and clinical outcome grades of the study population. No statistically significant differences were observed in baseline clinical characteristics between the two groups. Compared with their healthy counterparts, patients with AIS showed significantly increased levels of NRG-1 (*P* < 0.001; **Figure 1A**). Patients with AIS patients with an NIHSS score ≥ 6 at admission exhibited lower NRG-1 levels than those with an NIHSS score < 6 (*P* = 0.0393; **Figure 1B**). Furthermore, there was no statistically significant difference in NRG-1 levels between patients with AIS with hemorrhage transformation and those without (*P* = 0.4449; **Figure 1C**). No significant difference was found in long-term post-ischemia outcomes (NRG-1 levels) between patients with AIS with mRS ≤ 1 and those with mRS > 1 (*P* = 0.857), but we did observe a negative correlation between long-term post-ischemia outcome and 3-month mRS score (*r* = −0.449, *P* = 0.047; **Figure 1D** and **E**). These findings suggest that elevated serum NRG-1 levels in patients with AIS are closely associated with clinical features.

### Single-cell transcriptomic identification of Neuregulin-1-positive macrophage subtypes from the peripheral blood and clots of patients undergoing endovascular therapy

**Figure 2:**
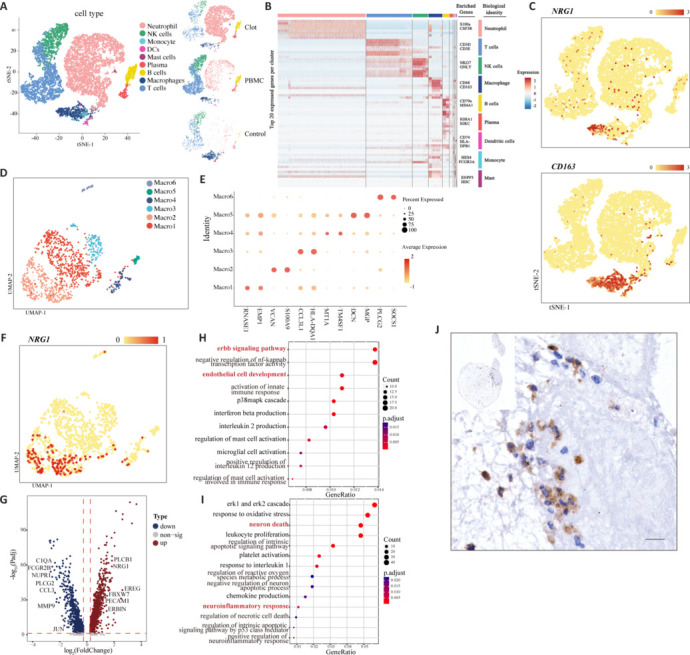
Single-cell transcriptomics identification of NRG-1^+^ macrophage subtypes from peripheral blood samples and clots from patients undergoing endovascular therapy. (A) Unsupervised clustering and tSNE identified nine major immune cell clusters from 14,163 cells in three samples. (B) Heatmap showing the relative expression of marker genes across CD45^+^ cell clusters. (C) NRG-1 is relatively high expressed in macrophages. (D, E) Macrophages were further re-clustered into six subtypes (Macro1–Macro6). (F) NRG-1 is highly expressed in the Macro2 subtype. (G) Volcano plot of transcriptomic differences between NRG-1-expressing and NRG-1-non-expressing cell subtypes. (H, I) Kyoto Encyclopedia of Genes and Genomes pathway analysis of expression differences between the Macro2 cluster and the other clusters. (J) Immunohistochemical staining for NRG-1 in clots from patients with acute ischemic stroke who received endovascular treatment. Scale bar: 1 μm. NRG-1: Neuregulin-1; t-SNE: t-distributed stochastic neighbor embedding.

To investigate the association between NRG-1 and peripheral immune cells, we examined paired samples of PBMCs and clots from patients with cardioembolic stroke undergoing endovascular therapy. CD45^+^ immune cells were isolated by FACS and subjected to scRNA-seq. Unsupervised clustering and t-distributed stochastic neighbor embedding (t-SNE) identified nine major immune cell clusters from 14,163 cells (**Figure 2A**). Based on established marker gene expression levels and unsupervised cell type annotation, these immune cell clusters were identified as B cells, dendritic cells (DCs), mast cells, T cells, natural killer (NK) cells, monocytes, macrophages, neutrophils, and plasma cells (**Figure 2B**). In addition, myeloid cells were abundant in the clots and PBMCs of patients with ischemic stroke. Notably, macrophages exhibited the highest level of NRG-1 expression, particularly in the peripheral blood, with lower expression detected in neutrophils (**Figure 2C**). Macrophages were further re-clustered into six subtypes (Macro1–Macro6), with NRG-1 expression predominately in the Macro2 subtype (**Figure 2D–F**). Differential gene expression analysis indicated upregulation of vascular-related genes (*EREG*, *PECAM1*, and *ERBIN*) and downregulation of neuroinflammatory genes (*LGMN*, *PLCG2*, and *APOE*) (**Figure 2G**). Kyoto Encyclopedia of Genes and Genomes (KEGG) analysis further highlighted enriched pathways, including endothelial development and ErbB signaling (upregulated) and neuroinflammatory responses (downregulated) (**Figure 2H** and **I**). Furthermore, immunohistochemistry (IHC) staining confirmed NRG-1 expression in thrombi (**Figure 2J**). These findings suggest that immune cells, particularly macrophages, may influence stroke pathology through NRG-1 secretion, elucidating their dual role in neuroinflammation.

### Neuregulin-1 is expressed in infiltrating myeloid cells in post-ischemic mice

**Figure 3:**
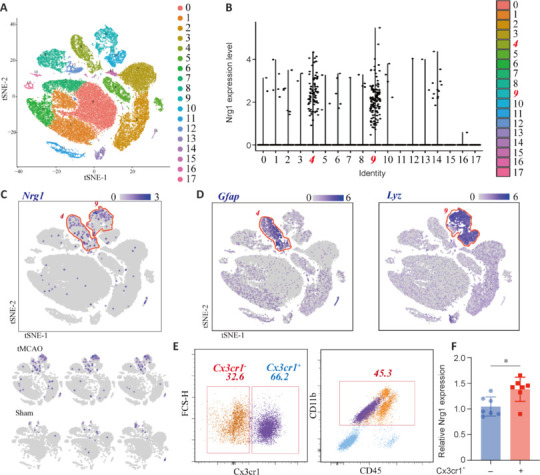
Bioinformatics detection of NRG-1 expression in infiltrating myeloid cells in post-ischemic mice. (A) Unsupervised clustering and tSNE identified 17 major cell clusters. (B) Violin plot showing that *Nrg1* is mainly expressed in the fourth and ninth cell subtypes. (C) *Nrg1* expression was increased in both cluster 4 and cluster 9 post-cerebral ischemic. (D) Cluster 4 was identified as astrocytes, based on the marker gene *Gfap*, and cluster 9 was identified as myeloid cells, based on the marker gene LYZ. NRG-1 expression increased post-ischemic stroke in both astrocytes and myeloid cells, especially in infiltrating myeloid cells. (E) FACS sorting of infiltrating cerebral myeloid cells post-ischemic stroke. (F) Quantitative reverse transcription–polymerase chain reaction analysis showing high expression levels of *Nrg1* in infiltrating myeloid cells. Data are represented as mean ± SEM. Statistical significance was identified using Student’s *t*-test. *P < 0.05. FACS: Fluorescence-activated cell sorting; FSC-H: forward scatter - height; NRG-1: Neuregulin-1; ns: not significant; tSNE: t-distributed stochastic neighbor embedding.

We have demonstrated elevated serum NRG-1 levels in patients with stroke and identified blood myeloid cells as the source using scRNA-seq. Although a prior study indicated that NRG-1 mitigates brain damage post-stroke (Liu et al., 2009), the involvement of infiltrating immune cells in this process remains unclear. To address this, we reanalyzed single-cell sequencing data from the brain tissue of sham and 24-hour middle cerebral artery occlusion (MCAO) mice retrieved from the GEO database (GSE174574). Consistent with a prior study (Zheng et al., 2022), 17 cell populations were identified through t-SNE (**Figure 3A**). NRG-1 expression was elevated in clusters 4 and 9, which were identified as astrocytes and myeloid cells, respectively (**Figure 3B** and **D**). NRG-1-expressing astrocytes (cluster 4) were observed in both the sham and tMCAO groups. In contrast, cluster 9, identified as myeloid cells, was significantly more prevalent in the tMCAO group than in the sham group, indicating that the infiltrating myeloid cells expressed NRG-1 (**Figure 3C**). To confirm NRG-1 expression in infiltrating myeloid cells, we sorted microglia (CD45^+^CD11b^+^CX3CR1^+^) and other myeloid cells (CD45^+^CD11b^+^CX3CR1^–^) from ischemic brain tissue 24 hours post-injury using FACS (**Figure 3E**). Quantitative PCR analysis demonstrated that infiltrating myeloid cells (CD45^+^CD11b^+^CX3CR1^–^) exhibited significantly elevated NRG-1 expression compared with microglia (CD45^+^CD11b^+^CX3CR1^+^) (*P* = 0.012), as shown in **Figure 3F**. These findings suggest that infiltrating myeloid cells, as well as glial cells, produce NRG-1 and exert protective effects after stroke.

### Macrophage-derived Neuregulin-1 reduces cerebral infarction volume and improves neurological deficits in mice with early-stage stroke mice

**Figure 4:**
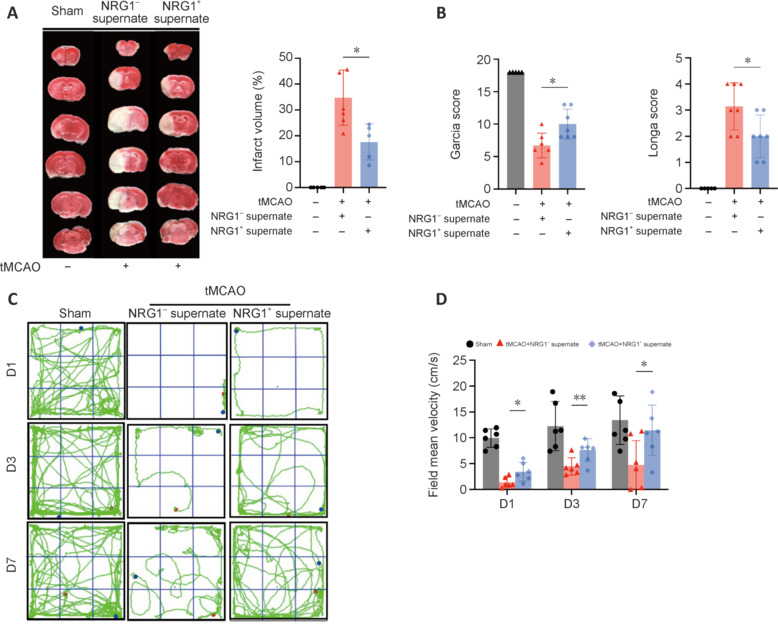
Macrophage-derived NRG-1 lowers cerebral infarction volume and improves neurological deficits in mice with early-stage stroke. (A) Macrophage-derived NRG-1 decreases infarct volume in mice subjected to tMCAO. White indicates the infarct region. (B) Macrophage-derived NRG-1 enhances behavioral prognosis in mice subjected to tMCAO, as assessed by Garcia and Longa tests. (C) Open field testing indicated that NRG-1 improves motor function in mice subjected to tMCAO. (D) Mean velocity of mouse locomotion in the open field test. Data are indicated as mean ± SEM. Statistical significance was determined using one-way analysis of variance with Tukey’s *post hoc* test. *P < 0.05; **P < 0.01; ***P < 0.001. NRG-1: Neuregulin-1; ns: not significant; tMCAO: transient middle cerebral artery occlusion.

NRG-1 (0.5 μg/kg) was administered intravenously to mice within 1 hour of tMCAO. Compared with PBS-treated controls, mice treated with NRG-1 exhibited significantly reduced brain infarct volume (*P* = 0.011) 3 days post-stroke (**Figure 4A**). Neurological function, as assessed by Longa and Garcia tests, improved following NRG-1 treatment (**Figure 4B**). Additionally, open-field testing revealed improved functional outcomes, specifically locomotor activity, in NRG-1-treated mice relative to controls (**Figure 4C** and **D**). These findings indicate that macrophage-derived NRG-1 mitigates ischemic damage and enhances early neurological recovery in a mouse model of tMCAO.

### Macrophage-derived Neuregulin-1 improves white matter damage, ameliorates neuroinflammation, and reduces long-term brain ischemic volume following cerebral infarction

**Figure 5:**
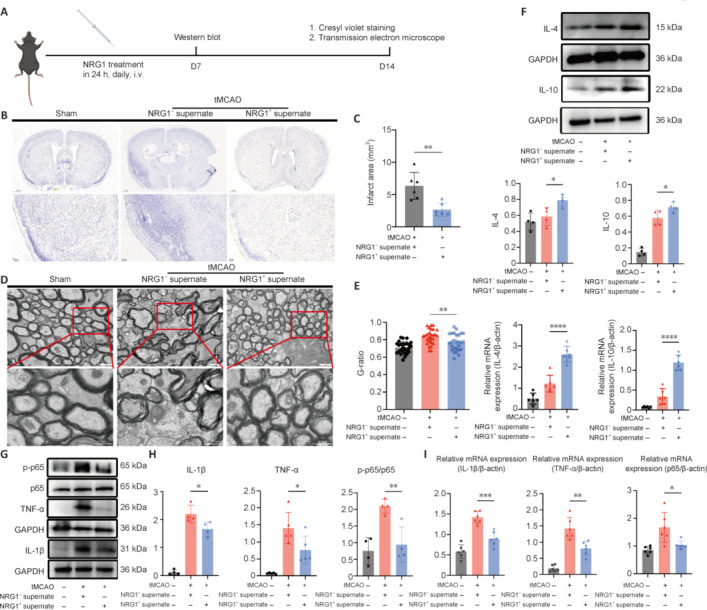
Macrophage-derived NRG-1 alleviates neuroinflammation and tissue damage in mice. (A) Experimental timeline. (B) Nissl staining of mouse brain sections. (C) Infarct volume. (D) Electron microscopy images of mouse brain myelin. Scale bars: 1 μm. (E) G-ratio of myelin in each group. (F) Western blot and quantitative reverse transcription–polymerase chain reaction analyses showing that expression of the anti-neuroinflammatory factors IL-4 and IL-10 was increased in mice subjected to tMCAO and treated with NRG-1. (G) Western blot analysis showing that macrophage-derived NRG-1 alleviated neuroinflammation by reducing TNF-α and IL-1β levels after tMCAO. (H, I) Quantitative western blot and quantitative reverse transcription–polymerase chain reaction analyses of the anti-neuroinflammatory effects of NRG-1 after tMCAO. Data are indicated as mean ± SEM. Statistical significance was determined using one-way analysis of variance with Tukey’s *post hoc* test. *P < 0.05; **P < 0.01; ***P < 0.001. GAPDH: Glyceraldehyde 3-phosphate dehydrogenase; IL: interleukin; NRG-1: Neuregulin-1; tMCAO: transient middle cerebral artery occlusion; TNF-α: tumor necrosis factor-α.

To investigate the effects of NRG-1 on white matter injury post-ischemia, we subjected mice to tMCAO and treated them with NRG-1 for 7 days. Nissl and Cresyl violet staining of brain sections 14 days post-stroke showed reduced ischemic brain volume in NRG-1-treated mice compared with the control mice (**Figure 5A–C**). Transmission electron microscopy (TEM) further revealed that NRG-1 ameliorated white matter injury, as demonstrated by improved G-ratio scores (**Figure 5D** and **E**). Western blot analysis showed that NRG-1 treatment suppressed p65 activation, suggesting an anti-inflammatory effect (**Figure 5F** and **G**). In addition, qPCR analysis also revealed decreased expression of TNF-α, IL-1β, and p65 (**Figure 5H** and **I**). These findings highlight the role of NRG-1 in white matter repair and its potential in reducing neuronal death, thereby contributing to better long-term outcomes after stroke.

### Macrophage-derived Neuregulin-1 enhances regional cerebral blood flow and promotes angiogenesis

**Figure 6:**
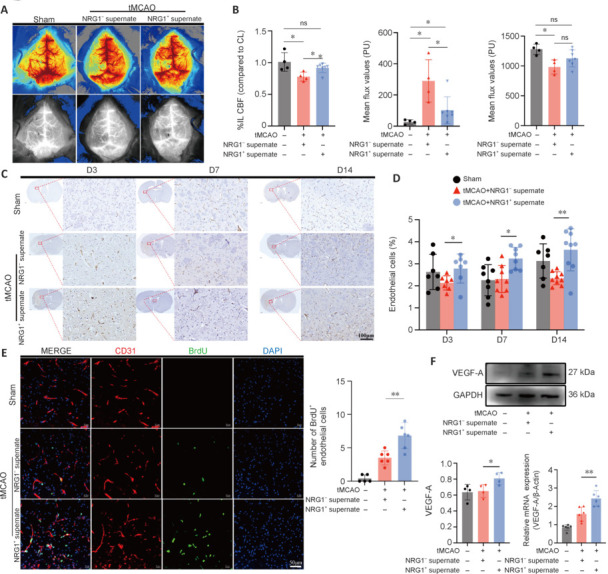
Macrophage-derived NRG-1 exerts protective effects by promoting angiogenesis in the peri-infarct area after tMCAO. (A) Laser speckle imaging of each group. (B) Quantification of CBF in each group. (C) Immunohistochemistry showing neovascularization in each group. (D) Quantification of the neovascularization shown in C. (E) Immunofluorescence showing CD31- and BrdU-positive areas and quantification of CD31^+^BrdU^+^ endothelial cells. (F) Western blot analysis showing VEGF-A expression in each group and quantitative reverse transcription–polymerase chain reaction analysis indicating that VEGF-A expression was elevated after administration of NRG-1. Data are presented as mean ± SEM. Statistical significance was identified using one-way analysis of variance with Tukey’s *post hoc* test. *P < 0.05; **P < 0.01. BrdU: 5-Bromo-2′-deoxyuridine; CBF: cerebral blood flow; CL: contralateral; DAPI: 4′,6-diamidino-2-phenylindole; GAPDH: glyceraldehyde 3-phosphate dehydrogenase; IL: ipsilateral; NRG-1: neuregulin-1; ns: not significant; tMCAO: transient middle cerebral artery occlusion; VEGF: vascular endothelial growth factor.

Considering that NRG-1 has been implicated in angiogenesis, we investigated its role in promoting neovascularization and regional CBF (rCBF) following ischemic stroke. Mice subjected to tMCAO mice received 50 mg/kg BrdU once daily for 7 days, after which laser speckle contrast imaging was performed to assess rCBF. NRG-1-treated mice demonstrated improved ipsilateral rCBF compared with controls (**Figure 6A** and **B**). Immunohistochemistry (IHC) revealed an increased number of endothelial cells in the peri-ischemic region 3, 7, and 14 days post-stroke (**Figure 6C** and **D**). Co-localization of BrdU with endothelial cells indicated enhanced neovascularization in NRG-1-treated mice (**Figure 6E** and **F**). These results indicate that macrophage-derived NRG-1 facilitates angiogenesis and improves rCBF after ischemic stroke.

### Macrophage-derived Neuregulin-1 promotes post-ischemic angiogenesis via the Akt/mTOR pathway

**Figure 7:**
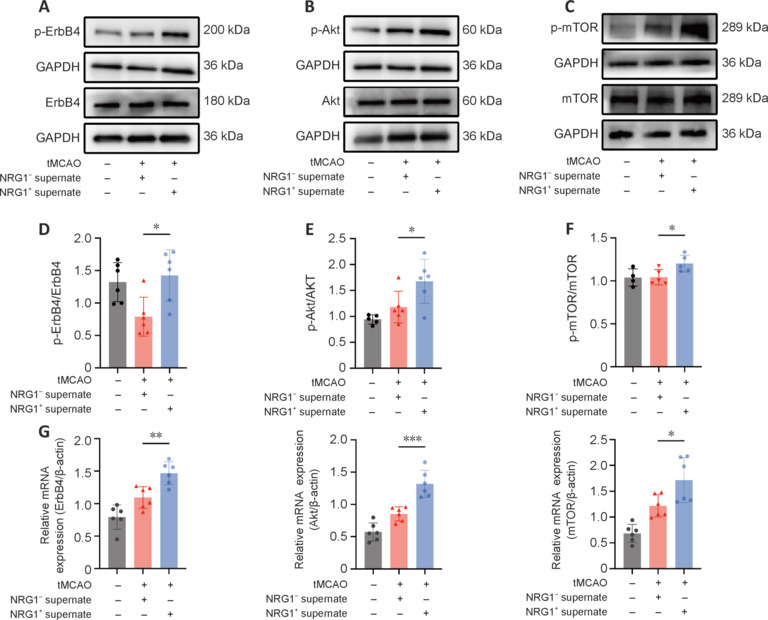
Macrophage-derived NRG-1 promotes post-ischemic angiogenesis through the Akt/mTOR pathway. (A–C) Western blot analyses showing ErbB4, Akt, and mTOR expression and phosphorylation in each group. (D–F) Quantification of the western blot data shown in A–C. (G) Quantitative reverse transcription–polymerase chain reaction analysis indicating ErbB4-Akt-mTOR signaling pathway activation in each group. Each group included at least three samples, with over three exposures for each sample. Data are denoted as mean ± SEM. Statistical significance was determined using one-way analysis of variance with Tukey’s *post hoc* test. *P < 0.05, **P < 0.01, ***P < 0.001. GAPDH: Glyceraldehyde 3-phosphate dehydrogenase; NRG-1: neuregulin-1; tMCAO: transient middle cerebral artery occlusion.

Western blot was used to identify the molecular signaling pathways through which NRG-1 exerts its effects. Analysis of brain tissue harvested 7 days after tMCAO showed that the ErbB4-Akt signaling pathway is activated to some extent after stroke, and that this activation signal is transmitted in the form of phosphorylation (**Figure 7**). These findings suggest that NRG-1 may activate the ErbB4 receptor through phosphorylation, which promotes downstream Akt-mTOR signaling and VEGF expression, ultimately enhancing angiogenesis. PCR analysis also confirmed activation of this pathway (**Figure 7**). Collectively, these results indicate that NRG-1 exerts its pro-angiogenic effects via the ErbB4-Akt-mTOR-VEGF axis.

## Discussion

Our findings revealed significantly elevated serum NRG-1 levels in patients with AIS. Additionally, using a mice tMCAO model, we identified peripheral macrophages as a key source of NRG-1 after AIS, highlighting their therapeutic potential in promoting angiogenesis and improving overall outcomes following mice cerebral ischemia.

NRG-1, a member of the epidermal growth factor family, has been previously recognized as a neuroprotective factor with significant potential for translation and clinical application (Noll et al., 2024). However, there is still a lack of sufficient research regarding its neuroprotective effects in the context of AIS. In this study, we found that, after AIS, peripheral monocytes and macrophages produce a certain amount of NRG-1, which promotes post-infarction injury repair.

The innate immune response, particularly macrophage polarization dynamics, critically modulates ischemic pathology through dichotomous cytokine secretion (pro-inflammatory *versus* resolution-phase mediators) (Shichita et al., 2014). Beyond their established role in cardiac angiogenesis through upregulation of pro-angiogenic factors such as insulin-like growth factor-binding protein 7 and hepatocyte growth factor, macrophages exhibit neurovascular crosstalk within the central nervous system (Denes et al., 2024; Landau et al., 2024). Recent evidence positions NRG-1 not merely as a macrophage-derived paracrine factor, but as an immunoregulatory nexus coordinating neuroprotection (Ryzhov et al., 2017). Notably, monocyte-derived macrophages serve as the principal immune source of plasma NRG-1, whose smaller isoform exhibits enhanced tissue diffusibility compared to other variants (Berrocal-Rubio et al., 2024). Our single-cell transcriptomic deconvolution of macrophage heterogeneity identified a distinct NRG-1-enriched subset (Macro2), mechanistically linking neuroimmune crosstalk to post-stroke vascular repair. This immunoregulatory axis establishes NRG-1^+^ macrophages as actionable therapeutic targets, forging a pathophysiology-informed translational paradigm for ischemic stroke intervention. To explore whether infiltrated myeloied cells expression NRG1, we reanalysis of single-cell RNA sequencing data from tMCAO mouse brain tissues in the GEO database revealed NRG1 expression patterns across cerebral cell populations following ischemic insult (Zheng et al., 2022). Compared with sham controls, NRG-1 was upregulated in ischemic brains, particularly within infiltrating myeloid cells and astrocytes. This expression profile aligned with our single-cell analysis of AIS patient, confirming myeloid-selective NRG-1 enrichment, thereby supporting the existence of an NRG-1-secreting macrophage subset.

Our mechanistic studies reveal that macrophages-derived NRG-1, acting through ErbB4 receptors, orchestrates post-ischemic vascular remodeling via synergistic VEGF/NRG1 signaling. Previous research in cardiovascular disease has already demonstrated that NRG-1 enhances myocardial glucose uptake and oxygen supply through activation of the PI3K-AKT signaling pathway (Fukazawa et al., 2003; Vaparanta et al., 2023). In the field of neurology, NRG-1 has been shown to lower neuroinflammation and protect neurons in animal models of spinal cord injury (Guan et al., 2019). Our findings extend this understanding by demonstrating that NRG-1 reduces infarct volume and enhances neurobehavioral recovery in a mouse model of ischemic stroke. Interestingly, recent studies have started to explore the role of NRG-1 in stimulating angiogenesis. Li et al. (2022) demonstrated that NRG-1 promoted stem cell differentiation into vascular cells through the PI3K-AKT pathway, while Peng et al. (2022) showed that NRG-1 expression was upregulated in hypoxic bone marrow stem cells, enhancing therapeutic efficacy under ischemic conditions. Similarly, studies have confirmed the role of NRG-1 in cardiac angiogenesis (Gui et al., 2018; Wu et al., 2018). Macrophage-derived NRG-1 appears to mediate cerebroprotection through IL-4/TGF-β-dependent suppression of acute neuroinflammatory responses (Abuzan et al., 2024). However, the potential of NRG-1 to promote angiogenesis post-stroke has been underexplored. In our study, using laser speckle contrast imaging, we observed improved cerebral blood flow recovery in NRG-1-treated mice. One study suggested that NRG-1 may exert its effects through the ErBb receptor (Li et al., 2007). Through western blot and PCR analyses, we demonstrated that NRG-1 exerts its effects through the ErbB4-AKT-mTOR pathway, promoting angiogenesis in the peri-infarct region. Therapeutic administration of macrophage-derived NRG-1 in tMCAO mice attenuated acute neuroinflammation while promoting peri-infarct vascularization and reducing white matter injury during chronic phases.

In tMCAO models, macrophage-derived NRG1 administration significantly enhanced peri-infarct vascular density and endothelial progenitor recruitment. Our findings identify NRG-1 as a pivotal regulator coordinating neuroimmune crosstalk with post-stroke vascular remodeling, while revealing macrophage heterogeneity as a critical determinant of angiogenic potential.

While current investigations predominantly focus on recombinant human NRG-1 or its exogenous cytoprotective effects on neuronal/glial populations post-ischemia, critical gaps persist in understanding the systemic regulation of NRG-1 within the neuroimmune-vascular triad. Our multi-modal study, employing single-cell transcriptomic profiling of thrombi and PBMCs from AIS patients combined with tMCAO mice models, identifies NRG-1^+^ macrophage subsets as immunological custodians. Mechanistically, macrophages-derived NRG-1 involves in post-stroke recovery through dual-axis regulation: 1) fostering angiogenesis via VEGF-dependent pathway and reducing white matter injury, and 2) resolving neuroinflammation. This work not only elucidates the long-term neuroprotective mechanism of NRG-1 underlying bridging vascular niche remodeling with immune homeostasis, but also highlights the therapeutic potential of macrophage-targeted NRG-1 delivery to overcome current neuroprotection bottlenecks.

Despite the promising findings, this study still had some limitations. First, the relatively small sample size of our study may limit the generalizability of the findings to the broader population. To validate our results, a larger, more representative case-control study is needed. Second, we used human clot samples for single-cell sequencing and validated these results using mouse models, which may have introduced some bias. Third, a major challenge in using NRG-1 for the treatment of cerebral ischemia is its limited capacity to effectively cross the blood–brain barrier. To address this issue, novel strategies—such as nanoparticle-based delivery systems, protein engineering, or other advanced technologies—are required to enhance the ability of NRG-1 to penetrate the blood–brain barrier and reach therapeutic concentrations within the brain. Although NRG-1 has shown angiogenic potential, it has also been associated with tumor growth, raising concerns about its safety in stroke applications. Moreover, further research is needed to determine safe and effective doses of NRG-1 for stroke treatment.

To conclude, in this study we identified peripheral macrophages as a key source of NRG-1 in the context of stroke. Our findings suggest that NRG-1 produced by peripheral macrophages promotes angiogenesis in the peri-infarct area, improving cerebral blood flow and overall outcomes in animal models. These findings highlight the therapeutic potential of macrophage-derived NRG-1 in stroke treatment and lay the groundwork for future clinical applications focusing on neuroprotection and vascular repair.

## Additional file:

***Additional Figure 1:*** Sample size calculated by G*Power software.

Additional Figure 1 Sample size calculated by G*Power software.

## Data Availability

*All relevant data are within the paper and its Additional file*.
