# Supplementary figures and images for: Macrophages-derived NRG-1 promotes angiogenesis after ischemic stroke via the Akt-mTOR pathway
Source: Neural Regen Res. 2025 Jun 19;21(7):3007–16. doi: 10.4103/NRR.NRR-D-24-01323 (PMC13378923; doi:10.4103/NRR.NRR-D-24-01323)

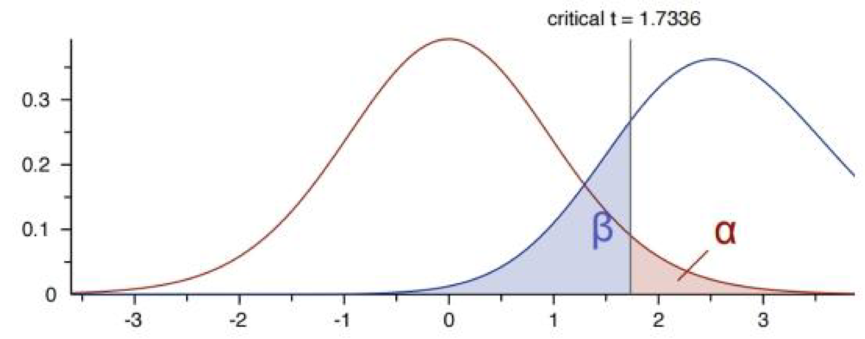

Supplement: Supplementary file 1 [file NRR-21-3007_Suppl1.tif]
